# Phosphatidylcholine in bile‐derived small extracellular vesicles as a novel biomarker of cholangiocarcinoma

**DOI:** 10.1002/cam4.5973

**Published:** 2023-04-25

**Authors:** Ryuta Muraki, Yoshifumi Morita, Shinya Ida, Ryo Kitajima, Satoru Furuhashi, Makoto Takeda, Hirotoshi Kikuchi, Yoshihiro Hiramatsu, Yusuke Takanashi, Yasushi Hamaya, Ken Sugimoto, Jun Ito, Kazuhito Kawata, Hideya Kawasaki, Tomohito Sato, Tomoaki Kahyo, Mitsutoshi Setou, Hiroya Takeuchi

**Affiliations:** ^1^ Second Department of Surgery Hamamatsu University School of Medicine Hamamatsu Japan; ^2^ Department of Cellular and Molecular Anatomy Hamamatsu University School of Medicine Hamamatsu Japan; ^3^ Department of Perioperative Functioning Care & Support Hamamatsu University School of Medicine Hamamatsu Japan; ^4^ First Department of Surgery Hamamatsu University School of Medicine Hamamatsu Japan; ^5^ First Department of Medicine Hamamatsu University School of Medicine Hamamatsu Japan; ^6^ Second Department of Internal Medicine Hamamatsu University School of Medicine Hamamatsu Japan; ^7^ Preeminent Medical Photonics Education & Research Center, Institute for NanoSuit Research Hamamatsu University School of Medicine Hamamatsu Japan; ^8^ International Mass Imaging Center Hamamatsu University School of Medicine Hamamatsu Japan; ^9^ Department of Systems Molecular Anatomy Institute for Medical Photonics Research, Preeminent Medical Photonics Education & Research Center Hamamatsu University School of Medicine Hamamatsu Japan

**Keywords:** bile, biomarker, cholangiocarcinoma, extracellular vesicle, phosphatidylcholine

## Abstract

**Background:**

Owing to the lack of definite diagnostic modalities, it is challenging to distinguish malignant cases of cholangiocarcinoma (CCA), which often causes biliary tract obstruction, from benign ones. Here, we investigated a novel lipid biomarker of CCA in bile‐derived small extracellular vesicles (sEVs) and developed a simple detection method for clinical application.

**Methods:**

Bile samples from seven patients with malignant diseases (hilar CCA = 4, distal CCA = 3) and eight patients with benign diseases (gallstones = 6, primary sclerosing cholangitis = 1, autoimmune pancreatitis = 1) were collected through a nasal biliary drainage tube. sEVs were isolated via serial ultracentrifugation and characterized using nanoparticle tracking analysis, transmission electron microscopy, and immunoblotting (with CD9, CD63, CD81, and TSG101). Comprehensive lipidomic analysis was performed using liquid chromatography–tandem mass spectrometry. Using a measurement kit, we further confirmed whether lipid concentrations could be used as a potential CCA marker.

**Results:**

Lipidomic analysis of bile sEVs in the two groups identified 209 significantly increased lipid species in the malignant group. When focusing on lipid class, phosphatidylcholine (PC) level was 4.98‐fold higher in the malignant group than in the benign group (P = 0.037). The receiver operating characteristic (ROC) curve showed a sensitivity of 71.4%, a specificity of 100%, and an area under the curve (AUC) of 0.857 (95% confidence interval [CI]:0.643–1.000). Using a PC assay kit, the ROC curve showed a cutoff value of 16.1 μg/mL, a sensitivity of 71.4%, a specificity of 100%, and an AUC of 0.839 (95% CI: 0.620–1.000).

**Conclusion:**

PC level in sEVs from human bile is a potential diagnostic marker for CCA and can be assessed by a commercially available assay kit.

## Graphical abstract

Owing to the lack of definite diagnostic modalities, it is challenging to distinguish malignant cases of cholangiocarcinoma, which often causes biliary tract obstruction, from benign ones. Phosphatidylcholine level in small extracellular vesicles from human bile is a potential diagnostic marker for cholangiocarcinoma and can be assessed by a commercially available assay kit.

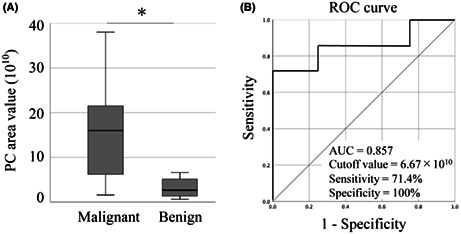

## 1 INTRODUCTION

Cholangiocarcinoma (CCA) is a type of biliary tract cancer that develops in the bile duct epithelium. Although considered rare, the incidence and mortality rate of CCA have been increasing worldwide over the past few decades.^1^, ^2^ Despite advances in CCA research, diagnosis, and therapy, the prognosis has not improved substantially with a continuing poor 5‐year survival rate (7–20%) in the past decade.^3^, ^4^, ^5^ The most frequent symptoms of CCA are biliary tract obstruction and jaundice.^6^ While benign stenosis can appear similar to CCA, it is difficult to distinguish benign from malignant bile duct stenosis.^7^ Although CCA diagnosis is based on multiple imaging techniques, such as CT, MRI, and endoscopic retrograde cholangiopancreatography (ERCP), these imaging modalities cannot definitively differentiate between benign and malignant stenosis. Other diagnostic techniques, including cytology and biopsy from the involved bile duct, have an unsatisfactory diagnostic accuracy and are challenging to perform.^8^, ^9^, ^10^ Therefore, accurate and straightforward biomarkers for CCA diagnosis are urgently needed.

Small extracellular vesicles (sEVs) play a vital role in intercellular crosstalk and have, in the last few years, gained interest as biomarkers for cancer detection.^11^, ^12^ According to size, EVs are classified into three groups: sEVs (40–150 nm), microvesicles (50–1000 nm), and apoptotic bodies (800–5000 nm).^13^ sEVs are secreted from various cells and are present in all body fluids, including blood, urine, and bile.^12^, ^14^, ^15^ Lipids are essential components of sEV membranes, and their composition is affected by the surrounding conditions.^14^ In particular, lipid in bile‐derived sEVs is of special interest, as bile is in direct contact with the cancerous cells in the biliary tract of a CCA patient.^16^, ^17^

We hypothesized that the lipid composition in bile‐derived sEVs was different between patients with CCA and those with benign disease. In this study, we aimed to identify a new biomarker for CCA by comparing the lipidomes of sEVs from the human bile of patients with malignant or benign conditions using liquid chromatography‐mass spectrometry (LC–MS/MS). Furthermore, to enable the use of this biomarker in clinical settings, we investigated whether the concentration of specific lipids could be simply measured with a commercially available assay kit.

## 2 MATERIALS AND METHODS

### 2.1 Patients and bile sample collection

Between April 2020 and March 2021, bile samples were collected from seven patients with malignant conditions (hilar CCA = 4, distal CCA = 3) and eight patients with benign conditions (gallstones = 6, primary sclerosing cholangitis = 1, autoimmune pancreatitis = 1) from the Hamamatsu University School of Medicine, Japan. All patients underwent ERCP, and a nasal biliary drainage tube, placed via a guidewire passed through the stricture, was used to diagnose or drain the bile samples. Brushing cytology and forceps biopsy were performed under radiologic imaging during ERCP. A cytological sample of bile was collected periodically from the next day of ERCP. The endpoint of bile sampling and submitted volume of bile depended on the handling criteria of personnel working in the internal medicine department. The bile collected just before the tube removal was analyzed to minimize cholestasis and inflammation. The samples were stored immediately at −80°C. In eligible patients of the malignant group, CCA was diagnosed using histology or cytology. The pathological features of CCA were determined in accordance with the TNM system, based on the eighth edition of the Union for International Cancer Control guidelines.^18^ Patients in the benign group were diagnosed with radiological and endoscopic features. They were followed up for more than 1 year after bile specimens were collected to exclude any early undiagnosed CCA. A patient who previously underwent endoscopic sphincterotomy was excluded because duodenal juice reflux into the bile might influence bile components. Written informed consent was obtained from all the patients. The study was approved by the ethical review board of our institution (approval number: 19–332) according to the ethical guidelines for clinical studies of the Japanese Ministry of Health, Labour and Welfare.

### 2.2 sEV isolation

To reduce viscosity, 1 mL of each bile sample was diluted with 11 mL of Dulbecco's phosphate‐buffered saline (D‐PBS). To pellet whole cells and debris, samples were centrifuged at 2000× *g* for 10 min at 4°C (Optima XE‐90, Beckman Coulter, rotor: SW41‐Ti). To remove high‐density subcellular structures and large apoptotic bodies, the supernatants were centrifuged at 10,000× *g* for 70 min at 4°C and filtered through a 0.22 μm filter (Merck Millipore). To remove residual supernatant, the filtrates were ultracentrifuged at 100,000× *g* for 70 min at 4°C, and the pellet was washed with fresh D‐PBS. Finally, the washed pellet was ultracentrifuged at 100,000× *g* for 70 min at 4°C and the pellet was resuspended in 100 μL fresh D‐PBS. The purified sEVs were stored at 4°C, and all subsequent processes were completed within a week of sEV extraction.

### 2.3 Transmission electron microscopy (TEM)

The sEV samples were mixed with 2% paraformaldehyde in phosphate buffer (pH 7.4) in a ratio of 1:1 and fixed for 5 min. A 10 μL sample of sEVs was placed on a copper grid (400 mesh, Cat. No. 2507, VECO), covered with 1.5% formvar support film, allowed to adsorb for 1 min, and then removed with filter paper. After washing once with distilled water, negative staining was performed with 2% uranyl acetate solution for 1 min, followed by drying. The samples were observed using a TEM (JEM‐1400 Plus; JEOL Ltd.) with an accelerating voltage of 80 kV, and captured with a CCD camera.

### 2.4 Western blot

Each sEV sample was homogenized in SDS sample buffer. Protein extracts (20 μg) were separated by 10% SDS‐PAGE, followed by electroblotting onto an Immobilon‐P polyvinylidene fluoride membrane (PVDF; Millipore). The membranes were blocked with 5% skim milk for 1 h at 24°C. Thereafter, the membranes were incubated with rat monoclonal anti‐CD9 (1:500; KMC8; Invitrogen), mouse monoclonal anti‐CD63 (1:1000; 10628D; Thermo Fischer Scientific Inc.), mouse monoclonal anti‐CD81 (1:1000; ab79559; Abcam), or rabbit polyclonal anti‐TSG101 (1:1000; ab30871; Abcam) primary antibodies for 1 h at 24°C. The membranes were washed using tris‐buffered saline with Tween 20 and incubated with anti‐rat IgG labeled with horseradish peroxidase (HRP; 1:3000; Jackson Laboratory Inc.), anti‐mouse IgG labeled with HRP (1:3000; Jackson Laboratory Inc.), anti‐mouse IgG labeled with HRP (1:5000; Jackson Laboratory Inc.), or goat anti‐rabbit IgG H&L (1:10,000; ab97051; Abcam) secondary antibodies for 1 h at 24°C. The primary and secondary antibodies were diluted in Can Get Signal Immunoreaction Enhancer Solution (Toyobo Life Science). After the final wash step, immunoreactive bands were visualized using the ECL Plus Western blotting Detection Reagents (GE Healthcare) and the Fusion FX7 system (Vilber Lourmat).

### 2.5 Nanoparticle tracking analysis (NTA)

The vesicle size and number were analyzed using a NanoSight NS300 system (NanoSight, Malvern Instruments). The samples were diluted 1:1000 in D‐PBS and loaded into a 1 mL disposable syringe. The Brownian motion of the particles in the solution was recorded five times for 60 s each, and the size and number of particles were analyzed using the NTA 3.1 software.

### 2.6 Lipid extraction

The modified Bligh and Dyer method was used for lipid extraction as described previously.^19^, ^20^ Phosphatidylcholine (PC) (12:0_12:0) (Avanti Polar Lipids) was used as an internal standard. In a glass tube, 40 μL of each sEV sample was mixed with 0.34 mL of methanol, 0.17 mL of chloroform, 0.14 mL of 0.322 M glacial acetate, and 0.182 nmol of PC(12:0_12:0). The solution was incubated for 10 min at 24°C, mixed with 0.17 mL of chloroform and 0.17 mL of 0.322 M glacial acetate, and allowed to separate into two phases. The lipid layer (lower phase) was separated from the aqueous layer by centrifugation at 720× *g* for 10 min at 24°C and transferred to a new glass tube. The sample was completely dried using the miVac Duo LV (Gen‐evac). The extracted lipids were dissolved in 30 μL of methanol, and 10 μL of the sample was used for lipid analysis.

### 2.7 Liquid chromatography–tandem mass spectrometry

We used a Q Exactive™ Hybrid Quadrupole‐Orbitrap™ Mass Spectrometer equipped with an electrospray ionization source coupled to an Ultimate 3000 system (Thermo Scientific). Briefly, 10 μL of the extracted lipid samples was injected and separated on an Acculaim 120 C18 column (150 mm × 2.1 mm, 3 μm; Thermo Scientific). Mobile phase, MS instrument, and Full‐MS mode conditions for quantification were the same as described previously.^20^

### 2.8 Lipid identification and quantification

To identify and quantify the lipid species, we used the LipidSearch™ software version 4.2.13 (Mitsui Knowledge Industry). Parameter settings for identification were as described previously.^20^ Alignment of the identified lipid species among the 15 patients was performed with a retention time tolerance of 0.6 min. The intensities of lipids recorded in the Xcalibur v3.0 software and monoisotopic peak area values of lipid species identified by LipidSearch™ software were normalized by dividing them by the area values of the internal PC control. Molecules that are annotated as redundant lipid ion names with different calculated *m/z* and retention times were regarded as independent structural isomers. In this study, the amount of lipid was reflected by the area value calculated by LC–MS/MS. The total lipid concentration in each sample was defined as the accumulation of the normalized lipid intensities.

### 2.9 PC quantification

The amount of PC was measured using a PC assay kit (Cell Biolabs Inc.), according to the manufacturer's instructions. Briefly, 10 μL of sEV sample and standard were added to 96‐well plates. Next, 100 μL of reaction reagent was added to each well. The plates were incubated at 37°C for 60 min in the dark. Plates were read with a fluorescence microplate reader at an excitation wavelength of 530–570 nm and an emission wavelength of 590–600 nm.

### 2.10 Statistical analyses

All continuous data were expressed as mean ± standard deviation or median (range, interquartile range). The Mann–Whitney *U* test or Student's t‐test was used to compare continuous variables. Pearson's chi‐squared test or Fisher's exact test was used to compare categorical variables. The average value of the normalized abundances that were obtained for each identified lipid between the two groups and the log_2_ (folding change) and ˗log_10_ (*p*‐value) were calculated and visualized using volcano plots. The optimal cutoff values to discriminate between the two groups were determined using receiver operating characteristic (ROC) curve analysis. The area under the curve (AUC) was calculated to validate the discrimination abilities of candidate lipids. Spearman's rank correlation analysis was used to validate the correlations among candidate lipid predictors. All calculations were performed using the SPSS Statistics version 26 software (IBM), and *p*‐values <0.05 were considered statistically significant.

## 3 RESULTS

### 3.1 Clinical characteristics of the patients

The clinical characteristics of the patients are presented in Table 1. All patients were of Japanese origin. C‐reactive protein (CRP) levels were significantly higher in the benign than in the malignant group, 1.6 [0.59–4.6] mg/dL versus 0.18 [0.07–1.70] mg/dL, respectively; *p* = 0.004 (Table 1). No significant difference was observed between the two groups regarding hepatobiliary function. All seven patients in the malignant group were diagnosed with no less than stage II CCA as the type of adenocarcinoma: four patients with hilar CCA and three with distal CCA (Table 2). The sensitivity of CCA diagnosis was 28.6% for the first cytological sample of bile and 33.3% for brushing cytology. The cumulative sensitivity was improved by repeated bile cytology to 57.1%. The number of bile cytological samplings ranged from 1 to 12 times (average, 5.1 times). The volume of bile submitted was 2 to 20 mL (average; 9.0 mL). Biopsy was performed in only 3 cases (2 cases of adenocarcinoma and 1 case of normal bile duct mucosa). Serum tumor markers also showed a low sensitivity; using carcinoembryonic antigen (CEA) levels (normal range: ≤5 ng/mL) and carbohydrate antigen 19–9 (CA19‐9) levels (normal range: ≤37 U/mL), 0% and 42.9% of CCA cases showed abnormally elevated levels, respectively.

**TABLE 1:** Clinical characteristics of the patients.

	Malignant (*N* = 7)	Benign (*N* = 8)	*p*‐value
Age	76 (56–79)	76 (38–82)	0.867
Sex (male/female)	7/0	8/0	—
Height (cm)	167 ± 5.10	167 ± 9.22	0.967
Weight (kg)	57.6 ± 9.05	58.0 ± 10.0	0.923
Body mass index	20.5 ± 2.55	20.9 ± 4.17	0.828
Total bilirubin (mg/dL)	1.1 (0.5–2.4)	1.4 (0.3–11)	0.779
Aspartate transaminase (U/L)	32 (22–109)	39 (9–397)	1.000
Alanine aminotransferase (U/L)	52 (30–187)	40 (16–695)	0.536
Alkaline phosphatase (U/L)	412 (287–1443)	561 (119–1673)	0.779
γ‐Glutamyl transpeptidase (U/L)	188 (51–381)	149 (36–516)	0.955
White blood cell count (/μL)	5473 ± 1378	5690 ± 1972	0.811
C‐reactive protein (mg/dL)	0.18 (0.07–1.70)	1.6 (0.59–4.6)	**0.004**

*Note*: Continuous data are presented as median (range) or mean ± standard deviation, whereas categorical data are shown as number of patients. Significant *p*‐value is in boldface.

**TABLE 2:** Oncological characteristics in the malignant group.

Case	Diagnosis	TNM classification	Cytology at first time^a^	Brushing cytology^a^	Repeated bile cytology^a^	Forceps biopsy	Serum CEA (ng/mL)	Serum CA19‐9 (U/mL)
1	Hilar cholangiocarcinoma	T2aN0M0 StageII	Positive	Negative	Positive	Not performed	4.9	25
2	Hilar cholangiocarcinoma	T2bN0M0 StageII	Positive	Positive	Positive	Not performed	3.6	333
3	Hilar cholangiocarcinoma	T2aN0M0 StageII	Negative	Negative	Negative	Adenocarcinoma	1.4	128
4	Hilar cholangiocarcinoma	T4bN0M0 StageIVA	Ngative	Positive	Positive	Adenocarcinoma	3.1	2661
5	Distal cholangiocarcinoma	T3bN0M0 StageIIA	Negative	Not performed	Negative	Not performed	2.3	14
6	Distal cholangiocarcinoma	T3aN1M0 StageIIB	Negative	Negative	Negative	Normal bile duct mucosa	2.2	24
7	Distal cholangiocarcinoma	T3aN1M0 StageIIB	Negative	Negative	Positive	Not performed	1.3	20

Abbreviations: CA19‐9, carbohydrate antigen 19–9; CEA, carcinoembryonic antigen; TNM, tumor, lymph node, metastasis.

^a^ Cytology including class IV and V was defined as positive.

### 3.2 sEV isolation from human bile

**FIGURE 1:**
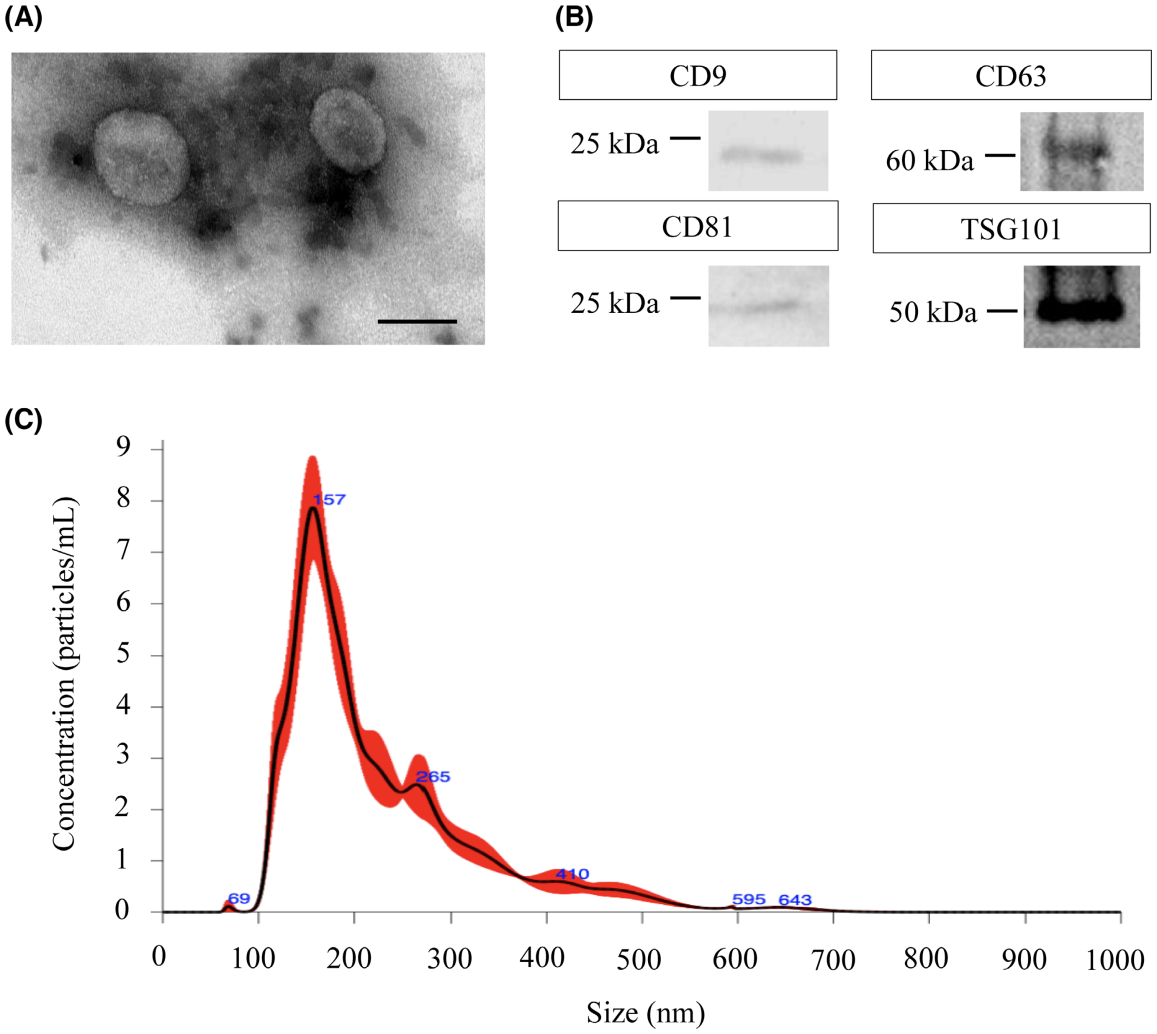
Characterization of sEVs present in human bile. (A) TEM image showing the typical round shape and morphology of sEVs. The scale bar represents 100 nm. (B) Western blot of the sEV protein markers (CD9, CD63, CD81, and TSG10). (C) Particle size and number distribution of sEVs were analyzed using NTA. Red area indicates the standard error ± 1 bars of the mean. NTA, nanoparticle tracking analysis; sEVs, small extracellular vesicles, TEM, transmission electron microscopy.

The presence of sEVs in human bile was confirmed using TEM, western blot, and NTA. TEM analysis of bile‐derived sEVs revealed a typical cup‐shaped and round morphology (Figure 1A). The presence of sEV markers CD9, CD63, CD81, and TSG101 was confirmed using western blotting (Figure 1B). The particle size and number were measured using NanoSight and were as expected for sEVs as observed in the typical particle size distribution chart (Figure 1C).

### 3.3 Quantification of sEVs by NTA between the malignant and benign groups

**FIGURE 2:**
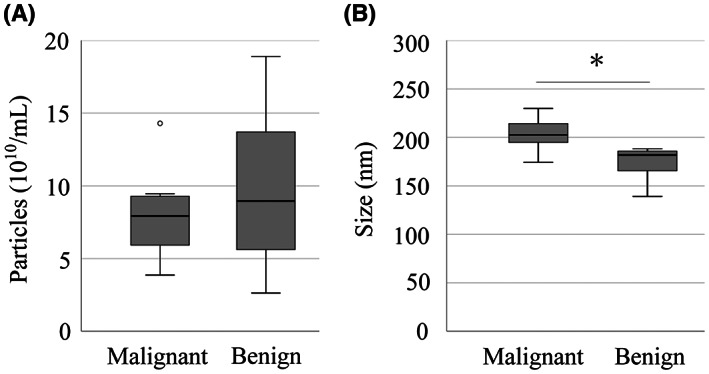
sEV quantification using NTA. (A) Box plot of sEV particle count and (B) Box plot of sEV particle size between the malignant and benign groups. Particles (10^10^/mL) indicate the number of particles derived from sEV fraction in 1 mL of bile sample. The asterisk indicates significant differences (*p* < 0.05). NTA, nanoparticle tracking analysis; sEV, small extracellular vesicle; TEM, transmission electron microscopy.

The average concentration of sEVs was 8.07 × 10^10^ and 9.76 × 10^10^ particles/mL in the malignant and benign groups, respectively (*p* = 0.499, Figure 2A). The size of sEVs in the malignant group (203 [174–230] nm) was significantly larger than that in the benign group (182 [139–188] nm, *p* = 0.002, Figure 2B).

### 3.4 Candidate lipids for predicting CCA

**FIGURE 3:**
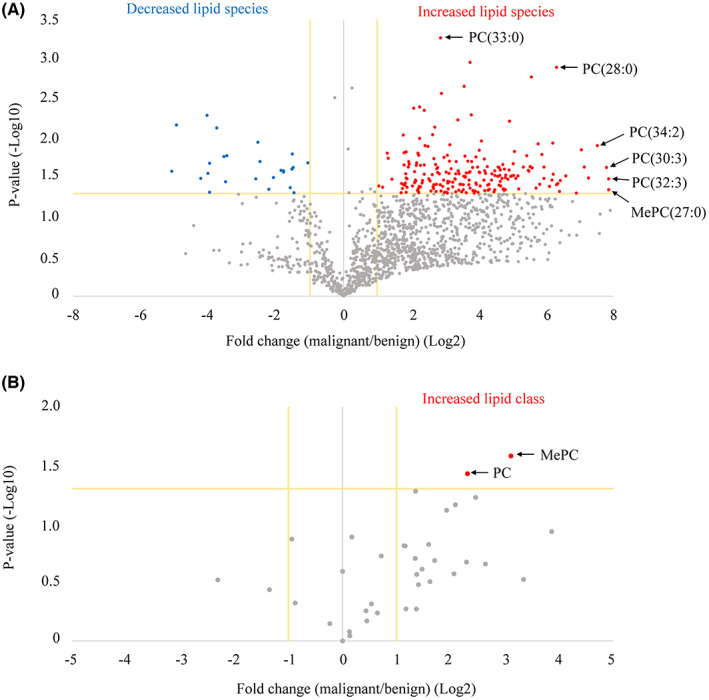
Lipidomic analysis using bile‐derived sEVs between the malignant and benign groups. (A) The volcano plot shows fold changes and *p*‐values of each lipid species in the malignant group compared to the benign group. The relative amount of 209 lipid species (red plots) increased (a fold change ≥2.0 and *p* < 0.05), while that of 25 lipid species (blue plots) decreased (a fold change ≤0.5 and *p* < 0.05) in the malignant group compared to the benign group. (B) The volcano plot shows the fold changes and *p*‐values of each lipid class in the malignant group compared to the benign group. The two increased lipid classes in the malignant group are indicated in red. MePC, methyl phosphatidylcholine; PC, phosphatidylcholine; sEV, small extracellular vesicle.

Bile‐derived sEVs from the two groups were separated using LC–MS/MS and compared by analyzing the data using the LipidSearch™ software. A total of 1525 lipid species identified in both groups were analyzed and visualized on a volcano plot (Figure 3A). We identified 234 lipid species that differed significantly in concentration between the two groups (fold change, ≥2.0 or ≤0.5; *p*‐values, <0.05). In the malignant group, 209 lipids were increased, and 25 lipids were decreased compared to the benign group (Table S1). The increased lipid species included lipids with various head groups; however, most were classified as PCs. Next, a volcano plot was constructed focusing on the lipid class. PC and methyl phosphatidylcholine (MePC) levels were found to be increased in the malignant group (Figure 3B, Table S2).

### 3.5 Comparison of lipid components between the malignant and benign groups

**FIGURE 4:**
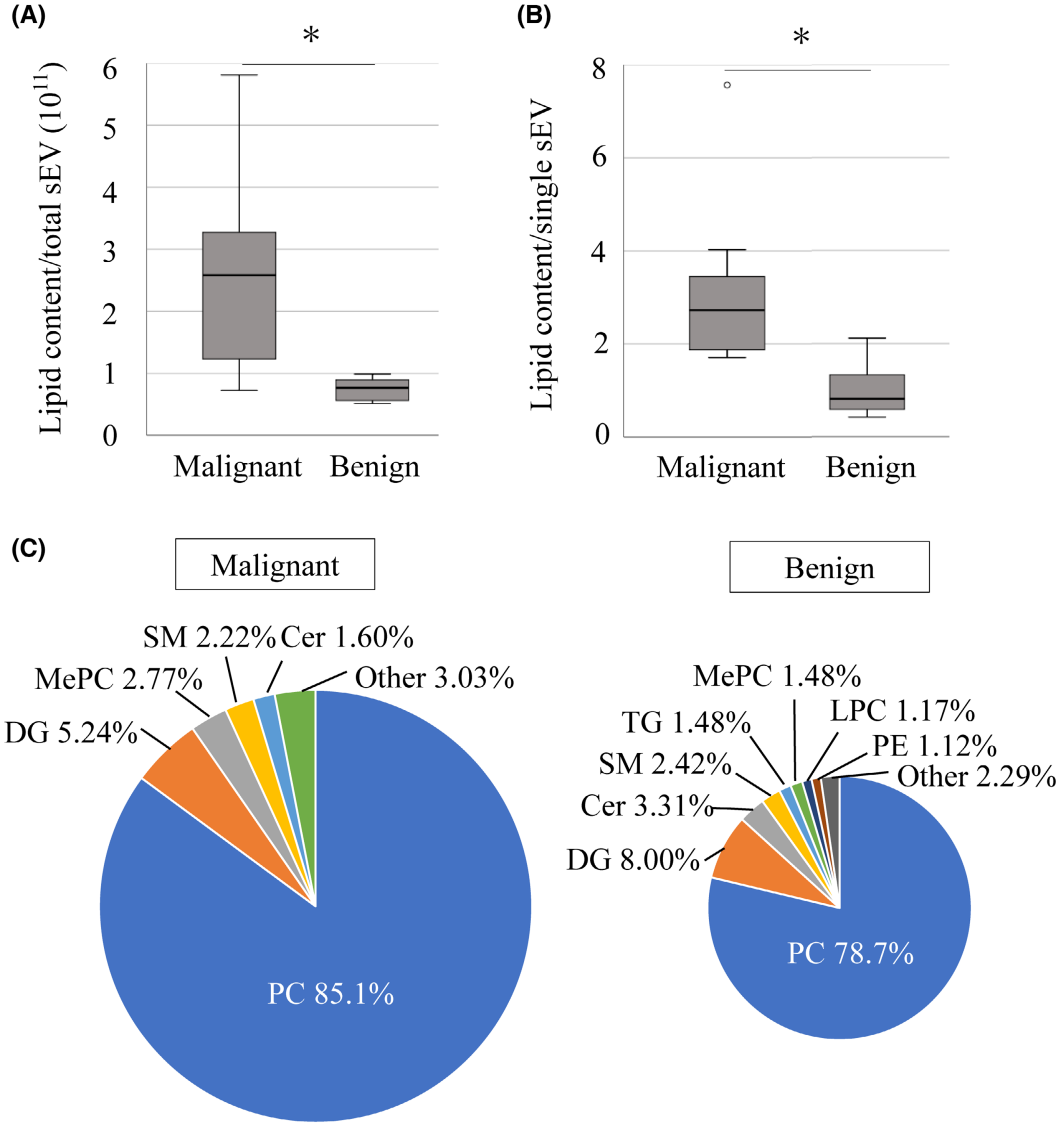
Comparison of lipid contents and distribution between the malignant and benign groups. (A) Box plot of lipid content per total sEVs. (B) Box plot of lipid content per each sEV. (C) Lipid distribution of major lipid classes. Asterisks indicate significant differences (*p* < 0.05). Ce, ceramides; DG, diglyceride; LPC, lysophosphatidylcholine; MePC, methyl phosphatidylcholine; PC, phosphatidylcholine; PE, phosphatidylethanolamine; sEV, small extracellular vesicle; SM, sphingomyelin; TG, triglyceride.

The total lipid level identified by LC–MS/MS for each patient was calculated by normalizing the intensities of the lipids. The average lipid content from total sEVs in the malignant group was 3.48 times higher than that in the benign group (*p* = 0.033; Figure 4A). When focusing on individual sEV, the average lipid content was also significantly higher in the malignant group (*p* = 0.002, Figure 4B). Next, we compared the distribution of the major types of lipids between the two groups. In both groups, PC was the major component of lipid in sEVs; 85.1% and 78.7% of total lipids were PCs in the malignant and benign groups, respectively (Figure 4C). In contrast, MePC was a minor component of lipid in sEVs (2.77% in the malignant group versus 1.48% in the benign group).

### 3.6 Diagnostic performance of bile sEVs

**FIGURE 5:**
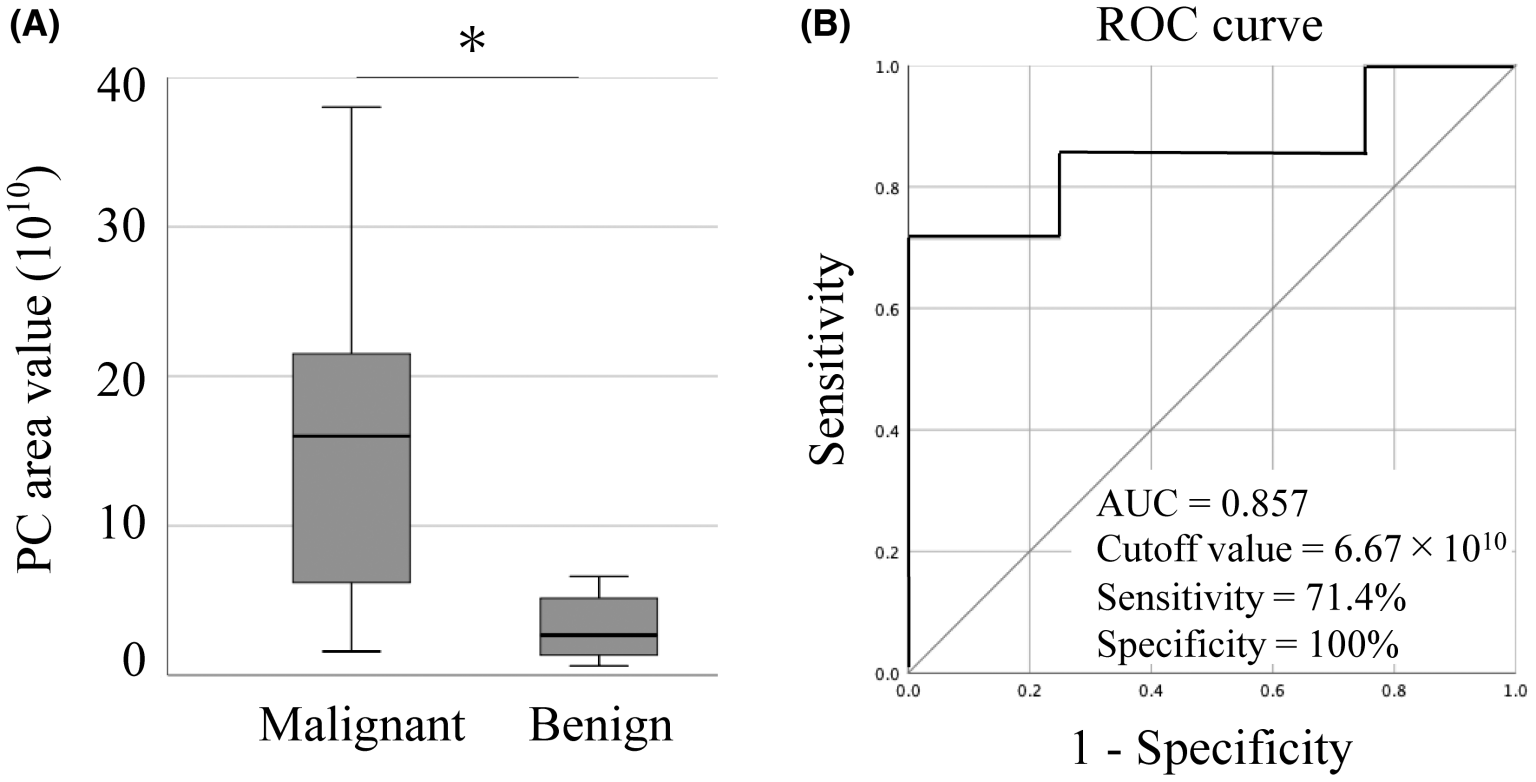
PC content in bile‐derived sEVs as a diagnostic parameter to discriminate between the malignant and the benign groups. (A) Box plot showing PC content based on area value. (B) ROC curve and ROC characteristics as defined by AUC for PC area values. The asterisk indicates significant differences (*p* < 0.05). AUC, area under the curve; PC, phosphatidylcholine; ROC, receiver operating characteristic; sEV, small extracellular vesicle.

**FIGURE 6:**
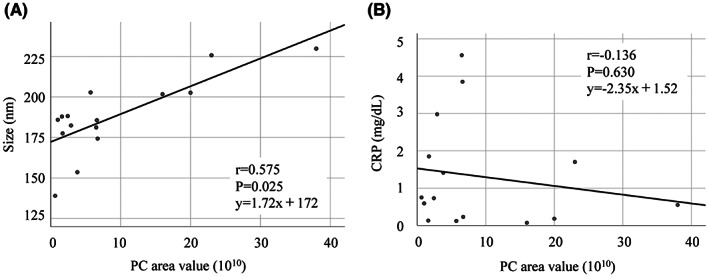
Correlation analysis between PC area value and sEV size or CRP level. (A) A positive correlation was observed between the sEV size and PC area value. (B) No correlation between CRP level and PC area value was detected. CRP, C‐reactive protein; *r*, correlation coefficient; PC, phosphatidylcholine; sEV, small extracellular vesicle.

We focused on the PC level in bile‐derived sEVs due to their higher concentration. The area value denoting PC content was significantly higher in the malignant group (4.98‐fold; 15.9 × 10^10^ ± 12.5 × 10^10^) than in the benign group (3.19 × 10^10^ ± 2.32 × 10,^10^ *p* = 0.037, Figure 5A). The ROC curve revealed a cutoff area value of 6.67 × 10^10^, a sensitivity of 71.4%, a specificity of 100%, and an AUC of 0.857 (95% confidence interval [CI]: 0.643–1.000; Figure 5B). The PC area values strongly correlated with the median sEV size (*r* = 0.575, *p* = 0.025, Figure 6A). In contrast, the PC area values did not correlate with CRP levels (*r* = −0.136, *p* = 0.630, Figure 6B).

### 3.7 A translational approach using a PC measurement kit

**FIGURE 7:**
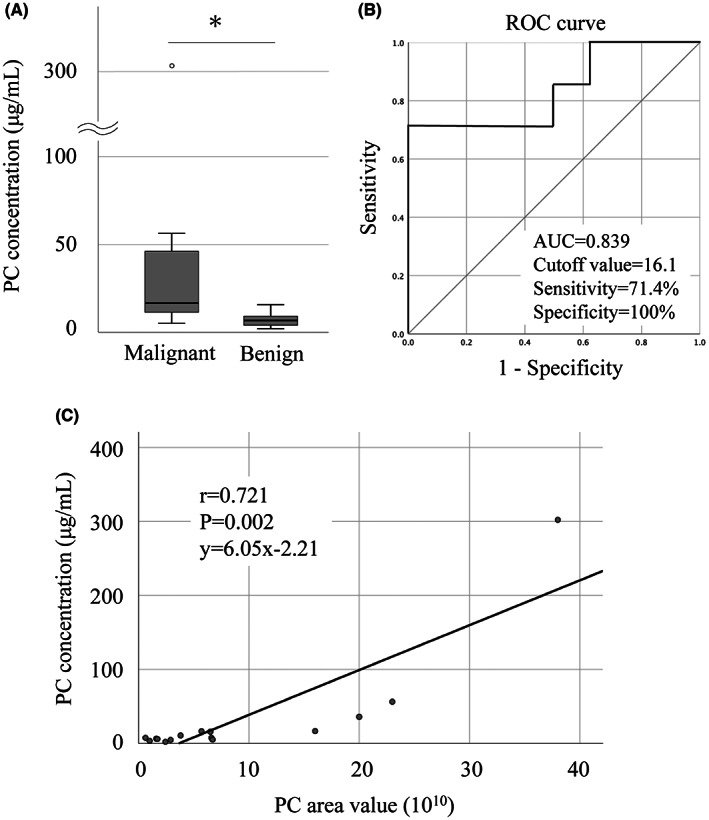
Diagnostic accuracy using a commercially available PC assay kit. (A) Box plot showing PC concentrations between the malignant and benign groups. PC concentration (μg/mL) indicates PC amount (μg) derived from sEV fraction in 1 mL of bile sample. (B) ROC curve and ROC characteristics as defined by AUC for PC concentration. (C) A positive correlation was detected between the PC area value and the PC concentration. The asterisk indicates significant differences (*p* < 0.05). AUC, area under the curve; *r*, correlation coefficient; PC, phosphatidylcholine; ROC, receiver operating characteristic; sEV, small extracellular vesicle.

Finally, we evaluated whether the amount of PC in bile‐derived sEVs could be measured using a commercially available PC assay kit for future clinical CCA diagnosis. The measured PC concentration was significantly higher in the malignant group than in the benign group, 2.45‐fold; 16.6 [5.10–302] μg/mL versus 6.78 [2.10–15.8] μg/mL, respectively; *p* = 0.029 (Figure 7A). The ROC curve revealed a cutoff value of 16.1 μg/mL, a sensitivity of 71.4%, a specificity of 100%, and an AUC of 0.839 (95% CI: 0.620–1.000; Figure 7B). The PC concentration strongly correlated with the PC area values (Figure 7C). Therefore, we propose that PC levels in bile‐derived sEVs are a useful biomarker for CCA diagnosis.

### 3.8 Subgroup analysis of the malignant and gallstone groups

The benign group included patients with gallstones (*N* = 6), primary sclerosing cholangitis (*N* = 1), and autoimmune pancreatitis (*N* = 1). Because these were completely different conditions, a subgroup analysis was performed comparing the results of the malignant group (*N* = 7) to those of the gallstone group (*N* = 6). PC levels shown by LC–MS/MS analysis and the measurement kit were higher in the malignant group than in the gallstone group (*p* = 0.047 and 0.051, respectively, Table S3). The ROC curve showed the PC area value and PC concentration as effective biomarkers for distinguishing the malignant group from the gallstone group as well as the benign group (Table S4).

## 4 DISCUSSION

To the best of our knowledge, this is the first report demonstrating that the PC level in human bile‐derived sEVs can be used as a novel biomarker of CCA. Additionally, the PC assay kit is easy to use for future clinical CCA diagnosis.

Human bile is a complex fluid produced and secreted by the liver and transported through the bile canaliculi and bile ducts. Its concentrations are influenced by the activity of hepatocytes and cholangiocytes.^21^, ^22^ Therefore, abnormal bile composition could be a reflection of a disease progression in the biliary tract.^21^, ^22^, ^23^, ^24^, ^25^ For instance, a previous study reported that the bile lipid component is a valuable diagnostic marker for CCA.^26^ In this report by Urman et al., a combination of some PCs, arachidonic acid (20:4), certain ceramides, and total triacylglycerols, increased their diagnostic accuracy. However, it required a complicated procedure, making it difficult to use in the clinical setting. Additionally, the quality and quantity of bile‐derived sEV components are more stable than those of whole bile, which possibly includes free‐floating cells.^17^ An analysis of whole bile, including free‐floating cells, has the potential for unpredictable biases and diagnostic results. Therefore, the analysis of narrowed targets from bile‐derived sEVs could be more useful for CCA diagnosis.

sEVs contain a lipid bilayer, which has an asymmetric distribution of lipid classes in the two plasma membrane leaflets. PC is one of the most common components of the outer leaflet.^27^ Lipids in sEVs not only have a structural role in their membranes but are also essential players in their formation and release to the extracellular environment. Unfortunately, the mechanism of changes in lipid composition in sEVs is not well understood.^28^ Additionally, the potential use of lipids in sEVs as biomarkers in the real world has not been explored in detail due to the limited number of studies on human samples. In this study, we performed a comprehensive lipidomic analysis of human bile‐derived sEVs, and upon focusing on the lipid class, PC and MePC were identified to be the statistically significant lipid classes elevated in the bile sEVs (Figure 3B). MePC, a derivative of PC, is a non‐inflammatory lipid, and its function has not been investigated in detail.^29^ Furthermore, using MePC as a CCA biomarker may be disadvantageous due to the low ratio of MePC among total lipid components (Figure 4C). Simple detection methods are required for establishing a useful biomarker in clinical settings. There is currently no commercially available assay kit to measure MePC levels; we therefore focused on evaluating PC levels as a CCA biomarker candidate.

Malignant tumors secrete large numbers of sEVs into the surrounding fluids, which are involved in tumor growth and differentiation.^30^ Bile is in direct contact with bile duct tumors, and bile‐derived sEVs could contain high concentrations of cancer biomarkers. Thus, the lipid content in bile‐derived sEVs is highly likely to contain a reliable biomarker candidate with high disease specificity. One study reported that the median size of bile‐derived sEVs from patients with pancreatobiliary cancer was higher than that of non‐malignant patients.^12^ Another study reported a significantly larger sEV size and a higher overall lipid amount in patients with prostate cancer than in healthy controls.^31^ Indeed, we found that both the median size and total lipid level of bile‐derived sEVs were significantly higher in the malignant group, mostly due to the higher PC concentration (Figures 4C and 6A). Because PC is one of the main components of the outer membrane of sEVs, we hypothesize that the larger the size of sEV, the greater the amount of PC.^27^ Additionally, the size of the sEV released by malignant tumors is larger than those released by normal cells.^12^ As a result, the CCA or adjacent cells (including hepatocytes and cholangiocytes) stimulated by the CCA are assumed to release a large amount of sEV, resulting in a large amount of PC.

In this study, we collected bile through a nasal biliary drainage tube just before tube removal to minimize the risk of cholestasis and cholangitis. A previous report described that bile samples collected during ERCP were affected by high bilirubin levels and inflammatory factors because the patients were at the beginning of therapy.^12^ In addition, alterations in the biliary constituents have been associated with the severity of jaundice and cholangitis.^32^, ^33^ It is also possible that inflammation could impact the concentration and nature of sEVs.^34^ Therefore, we decided to collect bile‐derived sEVs from patients without jaundice and cholangitis. In the present study, the CRP levels were significantly different between the two groups (median level; 0.18 mg/dL in the malignant and 1.6 mg/dL in the benign group). However, the CRP levels were generally low for all cases in this study. Furthermore, PC in the bile‐derived sEVs was not correlated with CRP levels (*r* = −0.136, *p* = 0.630, Figure 6B). The total bilirubin level was low and did not differ between the two groups, because of the post‐decompression biliary obstruction. Therefore, the possibility that cholestasis or cholangitis influenced our results could be excluded.

This study has some limitations. First, it was a single‐center study with a small sample size; thus, more extensive studies are required to verify our results. We are planning to conduct a multicenter trial based on this study. Second, coincidentally, this study did not include female cases. Larger studies including female cases are also needed in the future to verify the results of this study. Third, benign stenosis, which needs to be differentiated from CCA, was not included due to their small number. Finally, the mechanism of increased PC levels in bile‐derived sEVs remains unknown. The CCA itself, or the surrounding organs affected by the CCA, might secrete PC‐rich sEVs directly into the bile.

In conclusion, PC level in human bile‐derived sEVs is a promising CCA biomarker. Comprehensive lipidomic analysis using LC–MS is complicated and, therefore, difficult to perform during routine diagnostic procedures. Thus, the PC assay kit can simplify the complex procedure and is a promising diagnostic tool for CCA.

## AUTHOR CONTRIBUTIONS

**Ryuta Muraki:** Conceptualization (lead); data curation (lead); formal analysis (lead); funding acquisition (equal); methodology (lead); resources (equal); software (equal); visualization (lead); writing – original draft (lead); writing – review and editing (equal). **Yoshifumi Morita:** Conceptualization (equal); project administration (equal); supervision (equal); writing – original draft (equal); writing – review and editing (equal). **Shinya Ida:** Data curation (equal); investigation (equal). **Ryo Kitajima:** Data curation (equal); investigation (equal). **Satoru Furuhashi:** Data curation (equal); investigation (equal). **Makoto Takeda:** Data curation (equal); investigation (equal). **Hirotoshi Kikuchi:** Data curation (equal); funding acquisition (equal). **Yoshihiro Hiramatsu:** Data curation (equal); investigation (equal). **Yusuke Takanashi:** Formal analysis (equal); investigation (equal). **Yasushi Hamaya:** Resources (equal). **Ken Sugimoto:** Supervision (equal). **Jun Ito:** Resources (equal). **Kazuhito Kawata:** Supervision (equal). **Hideya Kawasaki:** Supervision (equal). **Tomohito Sato:** Supervision (equal). **Tomoaki Kahyo:** Supervision (equal). **Mitsubishi Setou:** Funding acquisition (equal); supervision (equal). **Hiroya Takeuchi:** Supervision (equal).

## ETHICAL APPROVAL STATEMENT

Written informed consent was obtained from all the patients. The study was approved by the ethical review board of the Hamamatsu University School of Medicine, Shizuoka, Japan (approval number: 19–332) according to the ethical guidelines for clinical studies of the Japanese Ministry of Health, Labour and Welfare.

## Supporting information


Table S1.

Table S2.

Table S3.

Table S4.

## Data Availability

The data that supports the findings of this study are available in the supplementary material of this article.
